# The prognostic value of neutrophil-to-lymphocyte ratio in patients with traumatic brain injury: A systematic review

**DOI:** 10.3389/fneur.2022.1021877

**Published:** 2022-10-24

**Authors:** Sherief Ghozy, Amr Ehab El-Qushayri, Joseph Varney, Salah Eddine Oussama Kacimi, Eshak I. Bahbah, Mostafa Ebraheem Morra, Jaffer Shah, Kevin M. Kallmes, Alzhraa Salah Abbas, Mohamed Elfil, Badrah S. Alghamdi, Ghulam Ashraf, Rowa Alhabbab, Adam A. Dmytriw

**Affiliations:** ^1^Department of Neuroradiology, Mayo Clinic, Rochester, MN, United States; ^2^Nuffield Department of Primary Care Health Sciences and Department for Continuing Education (EBHC Program), Oxford University, Oxford, United Kingdom; ^3^Faculty of Medicine, Minia University, Minya, Egypt; ^4^School of Medicine, American University of the Caribbean, Philipsburg, Sint Maarten; ^5^Faculty of Medicine, University of Tlemcen, Tlemcen, Algeria; ^6^Faculty of Medicine, Al-Azhar University, Damietta, Egypt; ^7^Faculty of Medicine, AlAzhar University, Cairo, Egypt; ^8^Drexel University College of Medicine, Drexel University, Philadelphia, PA, United States; ^9^Nested Knowledge, Saint Paul, MN, United States; ^10^Superior Medical Experts, Saint Paul, MN, United States; ^11^Department of Neurological Sciences, University of Nebraska Medical Center, Omaha, NE, United States; ^12^Neuroscience Unit, Department of Physiology, Faculty of Medicine, King Abdulaziz University, Jeddah, Saudi Arabia; ^13^Pre-Clinical Research Unit, King Fahd Medical Research Center, King Abdulaziz University, Jeddah, Saudi Arabia; ^14^Department of Medical Laboratory Sciences, College of Health Sciences, University of Sharjah, Sharjah, United Arab Emirates; ^15^Department of Medical Laboratory Technology, Faculty of Applied Medical Sciences, King Abdulaziz University, Jeddah, Saudi Arabia; ^16^Vaccines and Immunotherapy Unit, King Fahd Medical Research Center, King Abdulaziz University, Jeddah, Saudi Arabia; ^17^Neurointerventional Program, Departments of Medical Imaging and Clinical Neurological Sciences, London Health Sciences Centre, Western University, London, ON, Canada; ^18^Neuroendovascular Program, Massachusetts General Hospital and Brigham and Women's Hospital, Harvard Medical School, Boston, MA, United States

**Keywords:** mortality, neutrophil-to-lymphocyte ratio, prediction, traumatic brain injury, systematic (literature) review

## Abstract

Traumatic brain injury (TBI) places a heavy load on healthcare systems worldwide. Despite significant advancements in care, the TBI-related mortality is 30–50% and in most cases involves adolescents or young adults. Previous literature has suggested that neutrophil-to-lymphocyte ratio (NLR) may serve as a sensitive biomarker in predicting clinical outcomes following TBI. With conclusive evidence in this regard lacking, this study aimed to systematically review all original studies reporting the effectiveness of NLR as a predictor of TBI outcomes. A systematic search of eight databases was conducted according to the Preferred Reporting Items for Systematic Review and Meta-Analyses statement (PRISMA) recommendations. The risk of bias was assessed using the Quality in Prognostic Studies (QUIPS) tool. Eight studies were ultimately included in the study. In most of the studies interrogated, severity outcomes were successfully predicted by NLR in both univariate and multivariate prediction models, in different follow-up durations up to 6 months. A high NLR at 24 and 48 h after TBI in pediatric patients was associated with worse clinical outcomes. On pooling the NLR values within studies assessing its association with the outcome severity (favorable or not), patients with favorable outcomes had 37% lower NLR values than those with unfavorable ones (RoM= 0.63; 95% CI = 0.44–0.88; *p* = 0.007). However, there were considerable heterogeneity in effect estimates (*I*^2^ = 99%; *p* < 0.001). Moreover, NLR was a useful indicator of mortality at both 6-month and 1-year intervals. In conjunction with clinical and radiographic parameters, NLR might be a useful, inexpensive marker in predicting clinical outcomes in patients with TBI. However, the considerable heterogeneity in current literature keeps it under investigation with further studies are warranted to confirm the reliability of NLR in predicting TBI outcomes.

## Introduction

As one of the leading causes of death worldwide, traumatic brain injury (TBI) places a heavy burden on healthcare systems worldwide despite significant advancements in care (1). A recently published epidemiological study suggested that the age-adjusted mortality rate of TBI was 13–17 per 100,000 subjects (2). Furthermore, many reports have shown that the frequency of TBI mortality is 30–50% and that most cases involve adolescents or young adults (3–5). An additional socioeconomic burden on patients' families and community is a frequent consequence of major disabilities among survivors of TBI (1).

While primary brain damage is irreparable, secondary brain injury due to trauma-induced oxidative stress, ischemia, edema, and systemic response to inflammation can be remedied (1, 6–11). The inflammatory response following TBI is not fully understood, yet recent literature has demonstrated that such an inflammatory response might be prompted by damaged neuronal tissue. This damage triggers the production of proinflammatory cytokines and several angiogenic factors (12). This process further progresses to degeneration of tight junctions and protein extravasation (13). The uncontrolled release of inflammatory mediators, as well as the improper activation of endothelial cells, can affect the integrity of the blood-brain barrier (BBB), leading to fluid leakage to the interstitium and marked leukocytic infiltration (14). An *in vitro* study revealed that alteration of the BBB after the neuronal inflammatory response facilitates the migration of neutrophils into the injured area within the first hour of brain trauma, which may further affect the circulating white blood cells (WBCs) (15).

Assessment of peripheral WBCs, in terms of total and differential cell counts, is a straightforward and inexpensive test that provides a broad view of the entire systemic inflammatory process. Elevated WBC count was observed after delayed cerebral ischemia and deemed an independent risk factor for cerebral vasospasm after subarachnoid bleeding (16). Furthermore, the neutrophil-to-lymphocyte ratio (NLR) was proposed as a sensitive predictor of the inflammatory response in various neurological and non-neurological diseases such as stroke, Alzheimer's disease, and cardiovascular disorders (17–19). Moreover, it has been associated with poor clinical outcomes in certain types of cancer (20, 21). Similarly, reports have demonstrated that the NLR may serve as a sensitive biomarker in predicting clinical outcomes following TBI. Although conclusive evidence in this regard is lacking, these findings warrant further larger studies (22, 23). Therefore, this study aimed to systematically review all original studies reporting the effectiveness of NLR as a predictor of TBI outcomes.

## Methods

### Search strategy and study selection

We performed this systematic review and meta-analysis according to the Preferred Reporting Items for Systematic Review and Meta-Analyses statement (PRISMA) recommendations (24) using the AutoLit platform (Nested Knowledge, St. Paul, MN). We formulated the PICO question according to the following: population: patients with TBI; intervention: the neutrophil/lymphocyte sampling; comparator: healthy individuals/controls whenever available; outcome: the prognostic value of the NLR (e.g., mortality, morbidity, or improvement). After collecting the appropriate keywords for developing a search term (neutrophil^*^ OR lymphocyte^*^) AND ratio^*^ AND (Brain Injuries, Traumatic[MeSH] OR Trauma[Title]), we performed a systematic search for collecting relevant studies followed by a manual search from references to avoid missing any relevant papers. For databases not supporting MeSH terms, we used a combination of all possible keywords. The search was conducted on January 30, 2021, in eight databases: PubMed, Google Scholar, Embase, Scopus, Web of Science, The New York Academy of Medicine (NYAM), Virtual Health Library (VHL), and the System for Information on Grey Literature in Europe (SIGLE).

We included original studies that investigated the prognostic value of the NLR in patients with TBI. We excluded studies if they were (1) animal studies, (2) non-English articles, (3) non-original investigations such as protocols, reviews, posters, abstracts, and (4) case reports and case series of <5 patients. Title and abstract screening and full-text screening were done by at least two reviewers. The senior author was responsible for solving conflicts between the two reviewers.

### Data extraction

We conducted a pilot extraction of a few included studies for constructing a data extraction sheet. Then, two reviewers retrieved the necessary data from each of the included papers. The extraction sheet included the study design of the included papers, reference ID, demographic of the included population, outcomes of interest, and risk of bias tool. The senior author was responsible for solving conflicts between the two extractors.

### Risk of bias

Three independent reviewers evaluated the risk of bias in included studies. The risk of bias was assessed using the Quality in Prognostic Studies (QUIPS) tool (25, 26). Any discrepancy between the reviewers was solved by discussion.

### Statistical analysis

All data were analyzed using R software version 4.2.1. and the “meta” package. We did a priori sensitivity analysis comparing Standardized Mean Difference and Ratio of Means (RoM) computed results; in the case of similar results, RoM and its 95% confidence intervals (CI) were adopted due to easier interpretation of the results (27, 28). The analysis was conducted using a random-effects model due to considerable heterogeneity among the included studies. Heterogeneity was assessed with Q statistics and *I*^2^ test considering it significant with *I*^2^ value >50% or *P*-value <0.05 (29, 30). Due to the small number of the included studies (<10 per the analysis), neither Egger's regression test for assessing publication bias nor meta-regression was possible (31).

## Results

### Search results

**Figure 1:**
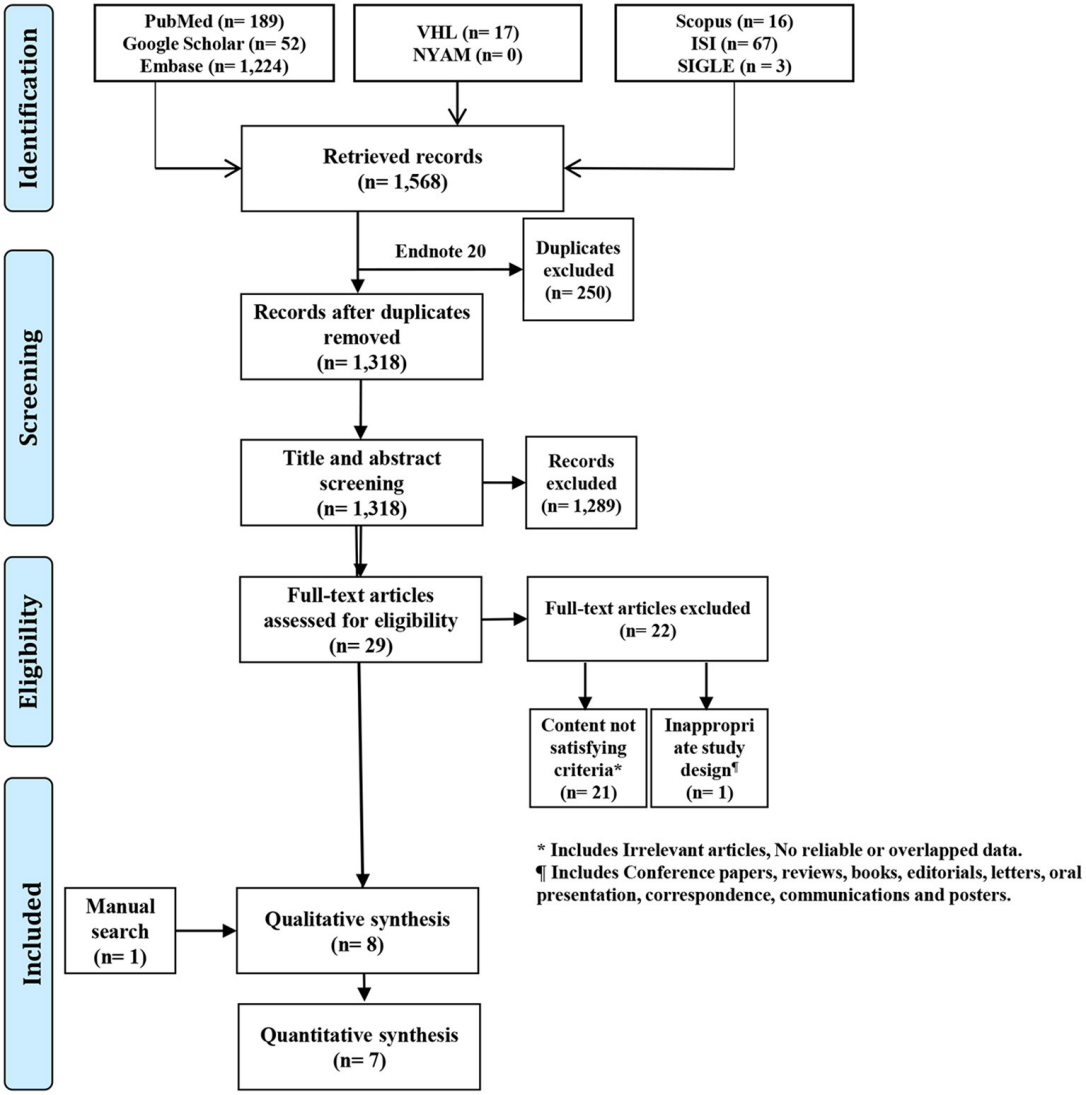
PRISMA flow diagram.

Following the combination of search results from all databases, a total of 1,568 records were retrieved. After removing duplicates using EndNote software (Clarivate Analytics, Philadelphia, PA), 1318 unique records were retained. The title and abstract screening filtered irrelevant papers to 29 records, which were further filtered by the full-text screening to seven relevant papers. We found one relevant paper using manual search methods to include a total of eight papers in the current study (Figure 1).

### Characteristics of the included studies

Details of the studies included in this systematic review are available in Table 1. Participants were included from several countries, including the United States (US), Turkey, China, Poland, and Australia. Of the eight included studies, seven were retrospective, and one employed a prospective study design. The timeframe of these studies was from January 1st, 2004, through December 31, 2017. The sample sizes ranged from 144 to 1291 patients. The seven retrospective studies used several severity measurements and scores. All retrospective studies used the Glasgow Coma Scale (GCS). Other metrics including Glasgow Outcome Scale-Extended Pediatric Version (GOS-E Peds), level of consciousness, post-traumatic amnesia, and Extended Glasgow Outcome Scale (GOSE). The prospective study by Akilli et al. (32) used the GCS, Acute Physiologic Assessment and Chronic Health Evaluation II (APACHE-II), and Sequential Organ Failure Assessment (SOFA) severity measurements. Generally, all included studies investigated the prognostic role of NLR. The retrospective study by Corbett et al. (33), which was based in Australia, specifically included patients who underwent decompressive craniectomy following severe TBI. Moreover, the retrospective study by Kimball et al. (22) based in the US specifically included patients aged 0 to 18. Three of the included studies (32, 34, 35) excluded patients with ages <18 and a history of hepatic or hematologic disease. Additionally, two of the eight studies (32, 35) screened out pregnant patients. Furthermore, we excluded studies that appeared as online only.

**Table 1:** Study and baseline characteristics of included studies and participants.

**Study and baseline characteristics**	**Kimball et al. (22)**	**Acar et al. (47)**	**Akilli et al. (32)**	**Chen et al. (34)**	**Zhao et al. (36)**	**Chen et al. (23)**	**Siwicka-Gieroba et al. (35)**	**Corbett et al. (33)**
**Study characteristics**								
Country	USA	Turkey	Turkey	China	China	China	Poland	Australia
Study design	Retrospective	Retrospective	Prospective	Retrospective	Retrospective	Retrospective	Retrospective	Restrospective
Time frame	January 01, 2007 December 31, 2017	January 01, 2013 December 31, 2014	January 01, 2013 August 10, 2013	January 2007 April 2012	December 2004 December 2017	January 2013 January 2017	NR	2004 - 2016
Sample size	*N =* 188	*N =* 200	*N =* 373	*N =* 688	*N =* 1291	*N =* 316	*N =* 144	*N =* 388
Inclusion critria	Age 0–18 years, isolated TBI, and at least one CBC panel with differential taken within 84 h of the time of injury	Patients with minor head trauma with isolated head trauma	Patients who had 2 of the 4 systemic inflammatory response syndrome criteria	Isolated head trauma, posttrauma GCS score 8 or less, time from injury to admission 6 h or less	CT scane-confirmed patients with TBI, CT signs of TBI, patients had to be >14 years of age. Patients had to be admitted within 6 h after injury	isolated head trauma, GCS score < 9; age > than 16, time interval from injury to admission < 24 h, at least 2 consecutive NLRs over a period of 3 days or more.	Adult patients with isolated severe TBI admitted to the intensive care unit (ICU)	Patients who underwent a decompressive craniectomy after severe TBI
Exclusion criteria	Severe comorbidities, prior neurological disease, anticoagulant, steroids, or immunosuppressants use, and prior systemic disease	Patients with GCS scores below 15, multiple traumas, chest pain, anemia, or chronic renal failure	Age < 18 years, pregnancy, hematologic disease, previous chemotherapy, blood transfusion, chronic hepatic disease, trauma, or poisoning.	Age < 18 years, time from injury to admission > 6 h, previous head trauma, ischemic or hemorrhagic stroke, antiplatelet, anticoagulants, steroids, immunosuppressant use presence of prior systemic diseases	Patients with TBI with traumatic injury to a body region other than the brain with an Abbreviated Injury Severity score > 3 and those with (34)penetrating brain injury	History of head trauma or other major diseases such as stroke, tumor, uremia, and heart failure. Missing data or loss to follow-up.	Patients aged < 18 years, pregnant women, patients with drug overdoses, patients with a history of neoplastic, cardiac, hepatic diseases, or renal diseases.	None
Severity measurement/scores	GOS-E Peds, LOC, GCS, PTA	CT scan findings	GCS, APACHEII, SOFA	GOS, GCS	GCS, GOS	GCS, GOS	GCS, GOSE	GOS, GCS
**Baseline characteristics of included participants**								
Age, years	9.49 ± 6.70a	35.25 ± 20.25a	74 (19)b	45.40 ± 14.85a	47.03 ± 16.88a	56 (43–63)b	48 (32–59)b	33 (22–49)b
Length of Hospital Admission, days	3, (1–48)b	NR	6.0 (9.1)b	NR	NR	18 (12–25.75)	NR	23 (13–45)b
**Gender**								
Male	118 (63 %)	151 (75.5 %)	203 (54.4 %)	557 (81 %)	982 (76.1 %)	256 (81.0 %)	118 (92 %)	310 (80 %)
**Type of injury**								
Skull fracture	NR	28 (14%)	NR	NR	299 (23.2 %)	NR	NR	NR
Diffusion axonal injury	NR	NR	NR	NR	46 (3.6 %)	NR	29 (20.1 %)	NR
Epidural hematoma	NR	27 (14%)	NR	NR	368 (28.5 %)	NR	NR	24 (6 %)
Subdural hematoma	NR	24 (12%)	NR	NR	378 (29.3 %)	NR	NR	NR
Subarachnoid hemorrhage	NR	15 (08%)	NR	NR	649 (50.3 %)	NR	NR	361 (93 %)
Intracerebral hematoma	NR	06 (03%)	NR	NR	860 (66.6 %)	NR	19 (13.2 %)	NR
**Clinical characteristics**								
Systolic arterial pressure, mm Hg	NR	NR	NR	130.54 ± 25.50a	NR	136 (123–150)b	NR	NR
Diastolic arterial pressure, mm Hg	NR	NR	NR	76.85 ± 15.53a	NR	79 (70–88)b	NR	NR
Mean arterial pressure, mm Hg	NR	NR	NR	95.12 ± 17.93a	NR	NR	NR	NR
Heart rate, beats/min	NR	NR	NR	85.7 ± 25.2a	NR	89.5 (79–109)b	NR	NR
Body temperature, °C	NR	NR	NR	36.86 ± 0.68a	NR	36.8 (36.6–37.2)b	NR	NR
Blood oxygen saturation, %	NR	NR	NR	95.35 ± 4.37a	NR	NR	NR	NR
Blood glucose level, mmol/L	NR	NR	NR	9.75 ± 3.41a	NR	8.7 (7.4–10.38)b	NR	NR
GCS Score on Admission	NR	NR	12 (8)b	5.95 ± 1.69a	11.21 ± 3.70a	7 (5–8)b	5 (3–6)b	8 (5–11)b
Survival	181 (96 %)	184 (92 %)^*^	NR	440 (64 %)	NR	NR	NR	NR

TBI, Traumatic brain injury; GCS, Glasgow Coma Sclae; GOS, Glasgow Outcome Scale; GOS-E Peds, Glasgow Outcome Scale-Extended Pediatric; APACHE II, Acute Physiology And Chronic Health Evaluation II; SOFA, Sequential Organ Failure Assessment score; LOC, Loss of consciousness; PTA, Post Traumatic Amnesia; NA, Not applicable; NR, Not reported; a, Mean ± SD; b, Median (IQR);

* number of dead is unknown.

### Characteristics of the included patients

Details of patient characteristics are in Table 1. The US retrospective study (15) had a mean patient age of 9.49 (SD: 6.70) years with a median length of stay of 3 (range: 1–48) days. The other five retrospective studies had mean ages ranging from 45.40 (14.85) to 47.03 (16.88) years and median ages ranging from 33 to 56 years. The Australia-based study had a median length of stay of 23 (IQR: 13–45) days (21). The prospective study by Akilli et al. (32) had a median patient age of 74 years with a median length of stay of 6.0 (IQR: 9.1) days. The gender distribution of the retrospective studies ranged from 63 to 92% male, with the prospective study having 54.4% male patients. Only two studies included survival data, which were 96% for the US study (15) in 2020 and 64% for Chen et al. (34) in 2018. The one prospective study had a median patient GCS score of 12 on admission (8). Chen et al. (34) conducted a retrospective study that included patients' clinical characteristics, including means of 130.54 (SD: 25.50) mmHg for systolic arterial pressure, 76.85 (SD: 15.53) mmHg for diastolic arterial pressure, 95.12 (SD: 17.93) mmHg for mean arterial pressure, 85.7 (SD: 25.2) beats/min for heart rate, 36.86 (SD: 0.68) °C for body temperature, 95.35% (SD: 4.37%) blood oxygen saturation, 9.75 (SD: 3.41) mmol/L for blood glucose, and 5.95 (SD: 1.69) GCS score on admission. The 2019 retrospective study by Chen et al. (23) demonstrated clinical characteristics, including medians of 136 (IQR: 123–150) mmHg for systolic arterial pressure, 79 (IQR: 70–88) mmHg for diastolic arterial pressure, 89.5 (IQR: 79–109) beats/min for heart rate, 36.8 (IQR: 36.6–37.2) °C for body temperature, 8.7 (IQR: 7.4–10.38) mmol/L for blood glucose, and 7 (IQR: 5–8) for GCS score on admission.

### Quality assessment of the included studies

QUIPS quality scores for risk of bias are presented in Table 2. Overall, the methodological quality of the included studies was satisfactory. Study participation and attrition were rated at a high risk of bias in one of the studies (34). All of the studies had a low to moderate risk of bias for prognostic factor measurement and outcome measurement. Furthermore, all of the studies were deemed acceptable with minimal risk of bias on statistical analysis and reporting.

**Table 2:** Risk of Bias of included studies.

**Study**	**1. Study participation**	**2. Study attrition**	**3. Prognostic factor measurement**	**4. Outcome measurement**	**5. Study confounding**	**6. Statistical analysis**
	**The study sample represents Population of interest on key characteristics?**	**The proportion of study sample providing outcome data is adequate?**	**The prognostic factor of interest is adequately measured in study subjects?**	**The outcome of interest is adequately measured in study subjects?**	**Important potential confounders are accounted for?**	**The statistical analysis is appropriate for the design of the study?**
Kimball et al. (22)	Yes	Not clear	Partly	Partly	No	Partly
Corbett et al. (33)	Partly	Partly	Partly	Partly	Not clear	Yes
Siwicka-Gieroba et al. (35)	Partly	Partly	Yes	Yes	No	Yes
Zhao et al. (36)	Yes	Not clear	Partly	Partly	Yes	Partly
Chen et al. (23)	Yes	No	Yes	Partly	Not clear	Yes
Chen et al. (34)	Yes	Partly	Yes	Yes	Not clear	Yes
Acar et al. (47)	Partly	Not clear	Yes	Partly	No	Yes
Akilli et al. (32)	Yes	Yes	Yes	Yes	Not clear	Yes

Studies were assessed under six domains of the QUIPS tool and given a rating (yes, no, partly, unclear; Hayden et al. (25, 26). A “yes” response indicates that the study has been designed and conducted to sufficiently limit the potential bias in that domain. An “unclear” or “partly” response arises when the answer to an item is not reported or is not reported clearly.

### NLR value and prognosis

**Figure 2:**
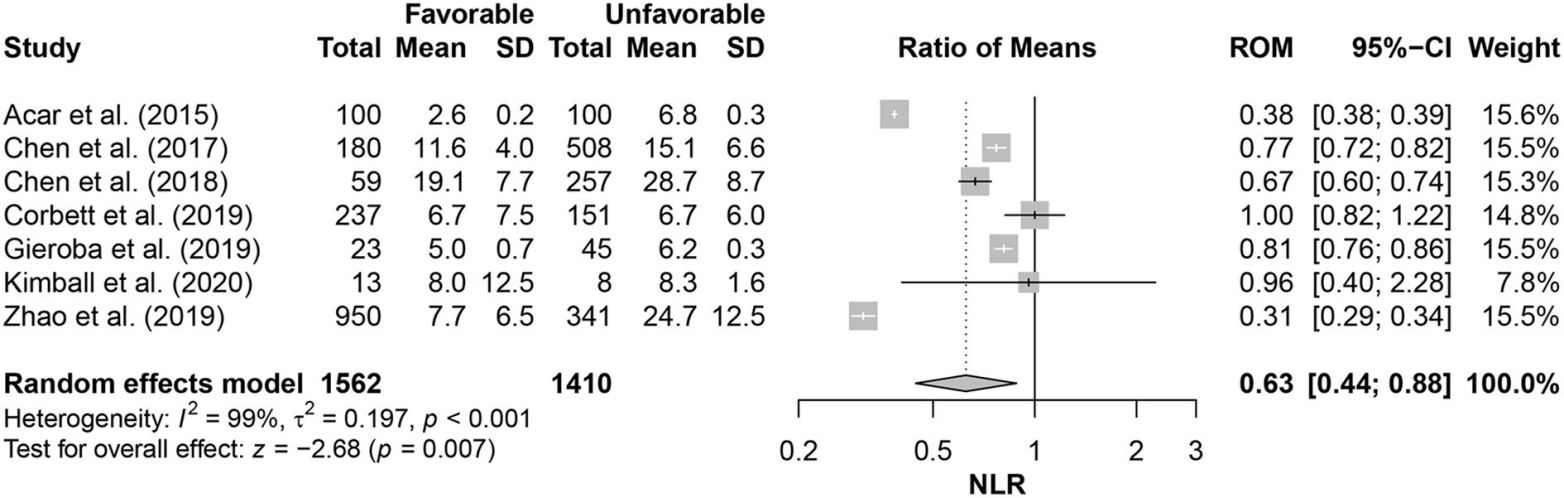
Comparison of neutrophil-lymphocyte ratio (NLR) in patients with favorable outcomes to those with unfavorable ones.

Relevant data of NLR values, outcome(s), outcome scale(s), and multivariate prediction model results (when applicable) are reported in Table 3. The outcome of severity was predicted with significant associations between NLR values and unfavorable outcomes in four studies, and mortality was predicted with significance in three studies. NLR significantly predicted unfavorable severity outcomes, as measured by GOS-E Peds, at 24 and 48 h with *p* = 0.004 and *p* = 0.003, respectively, in the study by Kimball et al. (22). There were no significant differences in the NLR of favorable and unfavorable outcomes in the study by the Corbett et al. (33); favorable outcomes had a median NLR value of 6 (IQR: 2–12), and unfavorable outcomes had a median NLR value of 6 (IQR: 3–11). This lack of significance remained in the multivariate prediction model (*p* = 0.870). The 6-month severity was predicted by the NLR with significance (*p* < 0.001) in the retrospective study by Zhao and colleagues (36). With the multivariate prediction model, significance remained (*p* < 0.001) with adjusted odds ratio (OR) of 0.91 (95% CI: 0.89–0.93). Both 1- and 12-day severity outcomes were predicted with significance in the 2019 study by Chen et al. (23), with *p* < 0.001 for both. On pooling the NLR values within studies assessing its association with the outcome severity (favorable or not), patients with favorable outcomes had 37% lower NLR values than those with unfavorable ones (RoM= 0.63; 95% CI = 0.44–0.88; *p* = 0.007). However, there were considerable heterogeneity in effect estimates (*I*^2^= 99%; *p* < 0.001) (Figure 2).

**Table 3:** NLR value and prediction according to worse outcomes.

**Source timeline**	**Outcome**	**Outcome score**	**NLR value**				**Multivariate prediction model of outcome**			
			**Favorable/** alive^*^		**Unfavorable/** dead^*^	**Significance**	**Measurement**	**Value**	**Significance**	**Variables included in the model**
Kimball et al. (22)		GOS-E 1 - 2	GOS-E 3 - 6	GOS-E 7 - 8					
< 12 h	Severity	GOS-E Peds	4.15 ± 5.87a	6.79 ± 8.42a	4.13 ± 4.94a	*P =* 0.38	NR	NR	NR	NR
24 h	Severity	GOS-E Peds	4.25 ± 3.43a	7.84 ± 4.27a	9.08 ± 4.55a	*P =* 0.004	NR	NR	NR	NR
48 h	Severity	GOS-E Peds	4.92 ± 3.05a	5.86 ± 2.98a	11.22 ± 1.95a	*P =* 0.003	NR	NR	NR	NR
72 h	Severity	GOS-E Peds	7.96 ± 12.50a	6.45 ± 3.58a	11.45 ± 2.85a	*P =* 0.80	NR	NR	NR	NR
Corbett et al. (33)
18 month	Severity	GOS	6 (2–12)b	NA	6 (3–11)b	*P =* 0.996	OR (95 % CI)	1.003 (0.972–1.035)	*P =* 0.870	IMPACT predicted risk; Hemoglobin, g/dl; Total white blood cells, × 109/L; NLR; Platelets, × 109/L; Fibrinogen, g/L; INR; aPTT, sec; DIC score; Glucose, mmol/L
Siwicka-Gieroba
et al. (35)
28 day	Mortality	NA	NA	NA	NA	*P < * 0.05	NR	NR	NR	NR
Zhao et al. (36)
6 month	Severity	GOS	07.68 ± 06.54a		24.71 ± 12.52a	*P < * 0.001	Adj OR (95 % CI)	0.91 (0.89 - 0.93)	*P < * 0.001	White blood cells, × 109/L; Neutrophil ratio; Lymphocyte ratio; NLR
Chen et al. (23)
Day 1	Severity	GOS	11.55 (08.62–14.11)b	NA	17.62 (13.08–20.89)b	*P < * 0.001	OR (95 % CI)	1.197 (1.125–1.273)	*P < * 0.001	Day 1 NLR; Admission GCS score
12 day (NLR peak)	Severity	GOS	18.62 (14.33–24.44)b	NA	27.34 (23.56–35.26)b	*P < * 0.001				
Chen et al. (34)
1 Year	Mortality	NA	13.75 ± 6.27a	NA	18.75 ± 7.76a	*P < * 0.001	Model I: OR (95 % CI)	1.141 (1.085–1.200)	*P < * 0.001	NLR; Deterioration; Mechanical ventilation
							Model II: OR (95 % CI)	1.158 (1.094–1.226)	*P < * 0.001	Temperature, °C; NLR; Deterioration; Mechanical ventilation
	Severity	GOS	11.60 ± 4.05a	NA	15.07 ± 6.63a	*P < * 0.001	OR (95 % CI)	1.100 (1.064–1.138)	*P < * 0.001	Age, y; NLR
Acar et al. (47)
Day 1	Abnormal CT scan	NA	2.61 ± 0.16a	NA	6.79 ± 0.55a	P ≤ 0.05	NR	NR	NR	NR
Akilli et al. (32)
Day 1	Mortality	NA	NR	NR	NR	NR	HR (95 % CI)	1.637 (1.110–2.415)	*P =* 0.01	NLR; APACHE II
180 day	mortality	NA	NR	NR	NR	NR	HR (95 % CI)	1.585 (1.136–2.213)	*P =* 0.007	NLR; APACHE II

a, Mean ± SD; b, Median (IQR); IQR, Interquartile range; HR, Hazard ratio; OR, Odds ratio; Adj, Adjusted; NR, Not reported;

* Favorable for severity/alive for mortality.

The multivariate predictive model retained outcome prediction significance (*p* < 0.001) in the 1-day category with an OR of 1.197 (95% CI: 1.125–1.273). The severity and 1-year mortality were both predicted with significance by NLR in the 2018 study by Chen et al. (34), with *p* < 0.001. This significance remained (*p* < 0.001) in the multivariate predictive model for the severity with 1.100 OR (95% CI: 1.064–1.138) and in both model I and model II for mortality with 1.141 OR (95% CI: 1.085–1.200) and 1.158 OR (95% CI: 1.094–1.226), respectively. Siwicka-Gieroba et al. (23) reported in their retrospective study that the 28-day mortality was significantly predicted (*p* < 0.05) by the NLR. In the prospective study by Akilli and colleagues (32), NLR significantly predicted both 1- and 180-day unfavorable mortality outcomes in the multivariate predictive model with hazard ratios (HRs) of 1.637 (95% CI: 1.110–2.415; *p* = 0.010) and 1.585 (95% CI: 1.136–2.213; *p* = 0.007), respectively. Supplementary Table 1 shows the parameters of the prediction models among the included studies.

## Discussion

**Figure 3:**
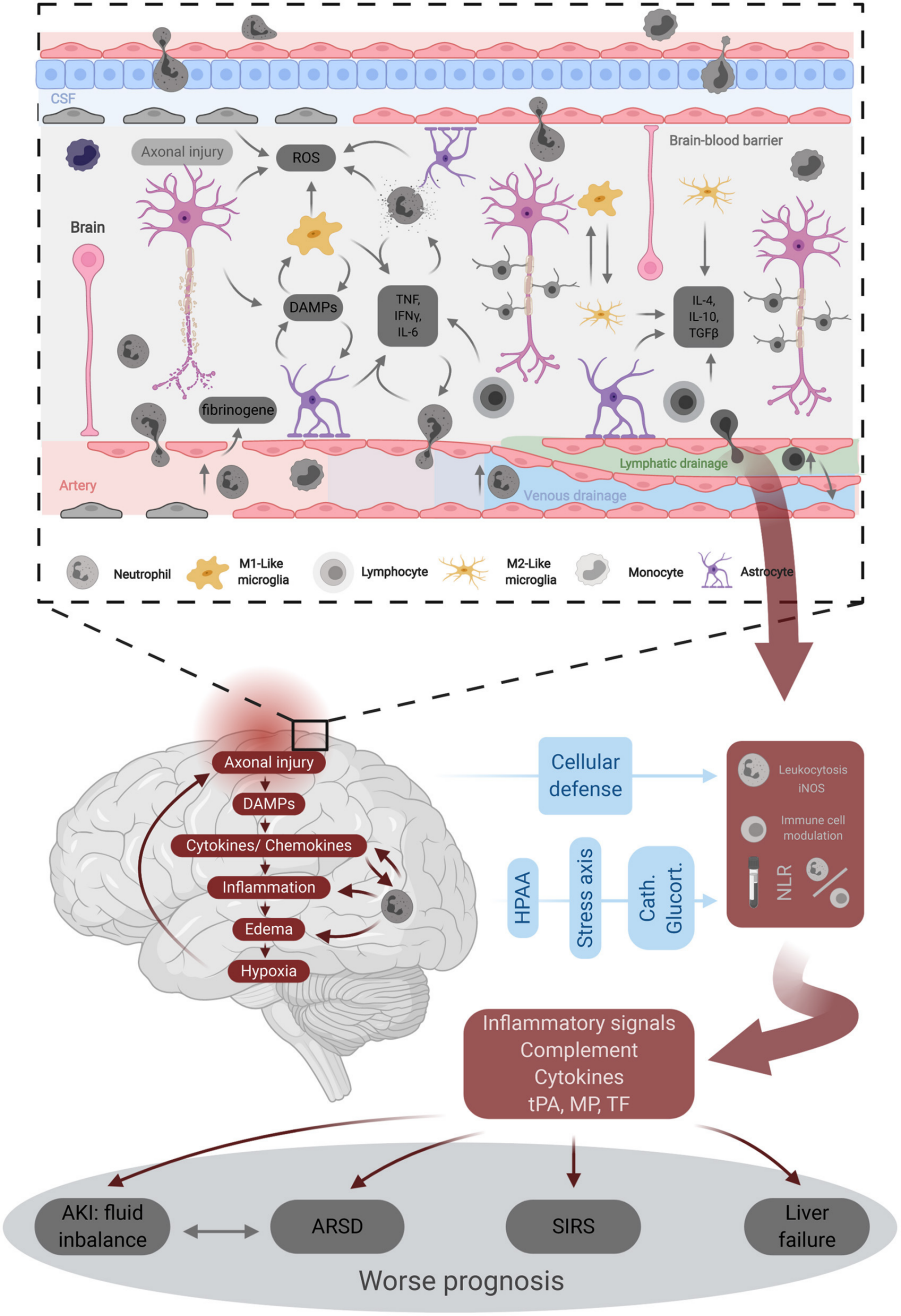
Underlying pathophysiology of traumatic brain injury. DAMPs, Damage-associated molecular patterns; iNOS, inducible nitric oxyde synthase; MP-TF, micro particles tissue factor; ROS, reactive oxygen species; SIRS, systemic inflammatory response syndrome; tPA, tissue plasminogene activator.

TBI affects millions of individuals worldwide on a yearly basis (37). This creates a taxing burden on healthcare systems in terms of financial resources or associated mortality. The pathophysiology of TBI is a highly complex process that relies on the primary brain injury resulting from the external injury (38) and the secondary injury that takes place within minutes of the primary one and can continue for several days afterward (37). This secondary injury is considered to be the net result of a cascade of cellular and molecular events and processes such as neuroinflammation, excitatory neurotoxicity, lipid peroxidation, edema, and mitochondrial dysfunction (6, 12, 39–41). As neuroinflammation has proved to play a critical role in the pathogenesis of TBI, the different components of such immune responses have been studied, including both pro-inflammatory and anti-inflammatory aspects (Figure 3) (23). Following TBI, a systemic immune response is mounted with significant changes in the inflammatory markers and different immune cells (42). Moreover, there is increasing evidence about the potential association between TBI and the progression of the neurodegenerative disease names chronic traumatic encephalopathy (CTE) (43). The condition is characterized by “an accumulation of abnormal hyperphosphorylated tau (p-tau) in neurons and astroglia distributed around small blood vessels at the depths of cortical sulci and in an irregular pattern,” with the condition is still under investigation (44, 45).

A retrospective study assessing TBI was completed on 144 patients with a GCS score lower than 8. The NLR was calculated at hospital admission and 6 days after the admission of patients at the intensive care unit. It found that the NLR at admission was significantly higher in patients who died compared with patients who survived at 4 weeks from admission (35). When the admission NLR was above 15.63, it was a predictor for mortality at day 28 after admission. The same study also demonstrated that a continuously high NLR during hospitalization was associated with poor clinical outcomes in patients with TBI. Subgroup analysis revealed that patients with diffuse axonal injury had a higher NLR compared with patients suffering from other complications of TBI such as cerebral edema or subarachnoid hemorrhage (35).

Age has been tested when assessing the reliability of the NLR in patients who are critically ill with TBI (46). Akilli et al. (32) performed a prospective observational cohort study on 373 older (mean age of 74) critically ill patients in the emergency department who were transferred to the intensive care unit. Admitted patients were assessed for NLR along with Acute Physiology and Chronic Health Evaluation II, SOFA, and GCS. Patients were followed up for evaluation of adverse outcomes and mortality at 6 months. The NLR was divided into four levels, with the lowest <3.48 and the highest more than 13.6. Multivariate Cox regression modeling showed that the NLR was an independent marker of both in-hospital and 6-month mortalities (32).

TBI in the pediatric population has also been explored in regards to NLR. In a retrospective 10-year study that encompassed 188 patients ranging from 0 to 18 years old, complete blood counts were used to calculate the NLR within 12 h of admission and again at 24, 48, and 72 h postadmission. Other information obtained from the records included GCS upon admission, post-traumatic amnesia, loss of consciousness, and the GOS-E Peds (22). Both the GCS upon admission and the presence of post-traumatic amnesia failed to show any significance in predicting clinical outcomes. Higher values of the NLR at 24 and 48 hours were associated with less favorable outcomes in pediatric patients suffering from TBI. Furthermore, patients who lost consciousness also had a significantly elevated NLR compared with patients who maintained consciousness (22).

In patients with minor head trauma, a retrospective study of 200 patients used computerized tomography (CT) scanning and blood markers to assess brain dysfunction in patients whose GCS were graded as 15 (47). Patients with normal CT scans served as the controls in this study. Blood values that were clinically significant included NLR and troponin-T. The NLR had a specificity of 90% when a cutoff value of 4.29 was implemented in assessing patients with detectable brain pathology on head CT in comparison with those who did not (47). This suggests that the NLR may have utility in patient assessment, not only in TBI but also in minor head trauma.

In a large study based in China, 855 patients (only 688 were included in the final analysis) who suffered from severe TBI were assessed for ~5 years. The initial NLR was calculated, as was the follow-up until 1 year after the TBI or death, whichever came first. Unfavorable outcomes were reported in 73.8% of patients at the 1-year follow-up of head trauma. In this group, an NLR upon admission for severe TBI was associated with a worse clinical outcome. Sensitivity and specificity of elevated NLR in predicting a negative outcome at the 1-year follow-up were found to be 60.2 and 71.1%, respectively (34).

A recent study was conducted to assess the prognostic utility of hematological markers after TBI. This study took place in Western Australia and involved 388 patients who underwent decompressive craniectomy after severe TBI (33). Unfavorable outcomes at 18 months were reported in 38.9% of patients and found to correlate with hematological abnormalities such as hemoglobin level, disseminated intravascular coagulation score, plasma glucose level, activated partial thromboplastin time, international normalized ratio (INR), and fibrinogen. Interestingly, an increased NLR was not associated with an increase in the incidence of unfavorable outcomes at 18 months post-decompressive craniectomy after severe TBI. After adjusting for the predicted risk of the International Mission for Prognosis and Analysis of Clinical Trials (IMPACT), the study concluded that the INR was the best blood parameter for 18-month survival in patients with severe TBI undergoing decompressive craniectomy (33).

The peak of the NLR in patients with severe TBI has been studied to assess its utility in predicting 1-year outcomes. A 4-year study of 316 patients reported that 81.3% experienced unfavorable clinical outcomes. The peak of NLR was found to be an independent predictor of unfavorable outcomes following severe TBI. Furthermore, the NLR on day one and the initial GCS score were found to be independently correlated with increased peak NLR (23). A large study was completed on TBI that involved 1,291 patients. The factors that were found to be independent predictors of negative outcomes after 6 months were age and admission GCS scores along with the presence of subdural hematoma, intraparenchymal hemorrhage, traumatic subarachnoid hemorrhage, or coagulopathy (36). Poor outcomes were associated with an increased NLR. When combined with certain standard prognostic factors such as age, GCS score, and coagulopathy, the NLR was reported to be capable of predicting the 6-month mortality more accurately (36).

Beyond the TBI, NLR was assessed in other neurological conditions, such as stroke. Khanzadeh et al. conducted a meta-analysis of 15 studies to evaluate using NLR to detect early poststroke infection (PSI) (48). They found significantly higher NLR levels in stroke patients with PSI compared to those without it (SMD = 0.98; 95% CI = 0.81–1.14; *p* < 0.001); however, the levels were comparable in terms of poststroke ventriculitis, sepsis, and urinary tract infections (48). In another meta-analysis of 3641 acute ischemic stroke patients -who received intravenous thrombolysis-, higher NLR levels were linked to higher odds of hemorrhagic transformation (OR = 1.33; 95 % CI = 1.14–1.56; *p* < 0.001) and poor 90-day functional outcome (OR = 1.64; 95 % CI = 1.38–1.94; *p* < 0.001) (49). In the same context, stroke patients with early neurological deterioration (END) had higher NLR levels than those without END (SMD = 0.73; 95% CI = 0.42–1.05; *p* < 0.001) (50).

Despite the limited evidence about NLR in TBI patients, our intellectual thoughts from the current evidence suggest that an increased NLR ratio correlates with poor prognosis in TBI patients. Nevertheless, the heterogeneity in the included studies, in terms of measurement intervals, follow-up points, and definitions of different outcomes, makes it impossible to draw any concrete conclusions. Further trials are needed to confirm the correlation between the NLR ratio and prognosis.

## Conclusions

A relatively inexpensive test, NLR can be easily and rapidly obtained in the emergency department. In this study, a high NLR at 24 and 48 h after TBI in pediatric patients was associated with worse clinical outcomes. In patients with minor TBI, the NLR was found to be an important prognostic marker when used in conjunction with head CT. NLR may be a useful predictor of the 6-month and 1-year mortalities. However, the overwhelming heterogeneity in current literature keeps the prognostic value of the neutrophil-to-lymphocyte ratio for TBI outcomes under investigation, and there are certainly more cost-effective and quick approaches to predict TBI outcomes, such as Glasgow Outcome Scale and Pupillary Light Reflex. Further studies are warranted to confirm the utility of NLR in predicting TBI outcomes.

## Data availability statement

The original contributions presented in the study are included in the article/Supplementary material, further inquiries can be directed to the corresponding author/s.

## Author contributions

All authors listed have made a substantial, direct, and intellectual contribution to the work and approved it for publication.

## Funding

This research work was funded by the Institutional Fund Projects under Grant No. (IFPDP-77-22).

## Conflict of interest

KK was employed by Nested Knowledge. The remaining authors declare that the research was conducted in the absence of any commercial or financial relationships that could be construed as a potential conflict of interest.

## Publisher's note

All claims expressed in this article are solely those of the authors and do not necessarily represent those of their affiliated organizations, or those of the publisher, the editors and the reviewers. Any product that may be evaluated in this article, or claim that may be made by its manufacturer, is not guaranteed or endorsed by the publisher.
